# Transcriptome and Metabolome Analyses Reveal the Molecular Mechanisms of *Albizia odoratissima*’s Response to Drought Stress

**DOI:** 10.3390/plants13192732

**Published:** 2024-09-29

**Authors:** Shuoxing Wei, Feng Gao, Zhihui Wang, Guoping Yin, Shizhi Wen, Hanbiao Ou, Zhiming Liu

**Affiliations:** 1 College of Forestry, Central South University of Forestry and Technology, Changsha 410004, China; 2 Guangxi Key Laboratory of Superior Timber Trees Resource Cultivation, Guangxi Forestry Research Institute, Nanning 530002, China; 20201200099@csuft.edu.cn (F.G.); gf20231101@163.com (Z.W.); gxlky1122@163.com (G.Y.); 3 Ping Ding Shan Industrial Technology Research Institute, Henan Academy of Sciences, Zhengzhou 450046, China

**Keywords:** *Albizia odoratissima*, key responsive genes, drought stress, transcriptome analysis, metabolome analysis

## Abstract

*Albizia odoratissima* is a deciduous tree species belonging to the family *Leguminosae*. It is widely distributed in the southern subtropical and tropical areas of China and has important ecological and economic value. The growth and metabolic processes of *A. odoratissima* are affected by drought stress, but the molecular mechanisms remain unknown. Therefore, this study investigated the physicochemical properties, gene expression, and metabolites of *A. odoratissima* seedlings under drought stress. The results show that, in leaves of *A. odoratissima* seedlings, drought stress reduced the moisture content, chlorophyll content, photosynthetic efficiency, superoxide dismutase (SOD) activity, and gibberellin (GA) and indoleacetic acid (IAA) contents while increasing the catalase (CAT) and peroxidase (POD) activities and malondialdehyde (MDA), proline, soluble sugar, and soluble protein contents. Within the CK5 (Day 5 of control group) vs. T5 (Day 5 of drought treatment), CK10 vs. T10, CK15 vs. T15, and CK20 vs. T20 groups (CK: control group; T: drought treatment), a total of 676 differentially expressed genes (DEGs) were upregulated and 518 DEGs were downregulated, and a total of 228 and 143 differential accumulation metabolites (DAMs) were identified in the CK10 vs. T10 and CK20 vs. T20 groups. These were mainly involved in the amino acid and alkaloid metabolism pathways in the leaves of the *A. odoratissima* seedlings. In the amino acid and alkaloid biosynthesis pathways, the relative expression levels of the *AoproA* (*Aod04G002740*, *ORTHODONTIC APPLIANCE*), *AoOAT* (*Aod07G015970*, *ORNITHINE-OXO-ACID TRANSAMINASE*), and *AoAOC3* (*Aod12G005010/08G003360/05G023920/08G003000/08G003010*, *AMINE OXIDASE COPPER CONTAINING 3*) genes increased, which concurrently promoted the accumulation of arginine, proline, piperine, cadaverine, and lysine. Furthermore, some key transcription factors in the response to drought were identified in the leaves using the weighted gene co-expression network analyses (WGCNA) method. These findings reveal that *A. odoratissima* seedlings respond to drought stress by improving the capacities of the antioxidant system and secondary metabolism.

## 1. Introduction

Drought is one of the major limiting factors for plant growth, development, yield, and distribution, seriously affecting the health of the global agroforestry industry [1]. Drought stress has important effects on plant morphology and structure, photosynthesis, antioxidant regulators, gene expression, and secondary metabolic processes. Drought stress induces the production and accumulation of intracellular reactive oxygen species (ROS) in plant cells and subsequently promotes lipid peroxidation and malondialdehyde (MDA) formation in the cells, resulting in membrane damage and structural incompleteness. Under drought conditions, the pigment content and photosynthetic efficiency in leaves significantly decrease, and, simultaneously, the transpiration rate decreases, as stomatal conductance is limited to reduce intracellular water loss, ultimately causing morphological changes in plants [2]. Meanwhile, drought stress promotes the biosynthesis and accumulation of soluble sugar, soluble protein, free proline, some secondary metabolites, and resistance hormones, thereby regulating plant growth and development [3]. Variations in these metabolites are controlled by the plant’s genes, suggesting that drought stress impacts metabolite content by regulating the expression levels of key genes in the biosynthesis and signal transduction pathways in plants [4]. The time and intensity of drought stress affect the cell division, physicochemical properties, gene expression, and metabolite accumulation in plants [5].

Plants develop numerous complex mechanisms in response to drought stress, such as phenotypic changes, physicochemical mechanisms, and molecular mechanisms, thereby improving drought resistance and adaptability [6,7,8,9]. In tobacco, drought stress has been found to increase the activities of superoxide dismutase (SOD), peroxidase (POD), catalase (CAT), ascorbate peroxidase (APX), and glutathione peroxidase (GPX), as well as the contents of soluble sugar, soluble protein, free proline, and glutathione (GSH), thereby reducing the damage of ROS to cells and ultimately increasing drought resistance [10]. Under drought stress, the concentrations of auxin, cytokinin, and gibberellic acid decrease, while the concentrations of abscisic acid (ABA), brassinosteroid (BR), ethylene (ET), jasmonic acid (JA), salicylic acid (SA), and strigolactone (SL) increase, thereby regulating homeostasis and improving drought resistance in plants [11]. Furthermore, drought stress increases the relative expression levels of the *MALATE DEHYDROGENASE* (*MDH*), *PYRUVATE DEHYDROGENASE* (*PDH*), *delta(1)-PYRROLINE-5-CAEBOXYLATE SYNTHETASE* (*P5CS*), *HEAT SHOCK PROTEIN* (*HSP*), and *CYTOCHROME P450* (*CYP*) genes, which are related to resistance and metabolism. This subsequently impacts the biosynthesis and accumulation of glucose, fructose, phenylpropanoids, carotenoids, zeaxanthin, monoterpenes, 4-hydroxycinnamic acid, and ferulic acid, which are involved in the pathways of the tricarboxylic acid cycle, glycolysis, glutamate-mediated proline biosynthesis, sucrose and starch metabolism, tyrosine metabolism, phenylalanine metabolism, phenylalanine biosynthesis, and secondary metabolism. Finally, this leads to the regulation of anatomical structures, physicochemical processes, and secondary metabolic processes, eventually improving the resistance and gene expression of the plants [12,13,14,15,16,17,18,19].

*Albizia odoratissima* is a diploid (2n = 26) deciduous tree species belonging to the family *Leguminosae*. *A. odoratissima* exhibits rapid growth, good wood properties, resistance to barrenness, and a strong renewal ability; therefore, it is cultivated at a large scale in Guangxi, Hainan, and other regions in China [20]. *A. odoratissima* has significant economic value because of its density, durability, stability, and resistance to decay and insect infestation, and it is widely used in high-quality furniture manufacturing, construction, and other industries [21,22]. Meanwhile, *A. odoratissima* also has significant ecological value as a top-ranking nitrogen-fixing tree species owing to its rhizobia, and it plays important roles in soil and water conservation and carbon sink increase. Moreover, *A. odoratissima*, as a native timber species, exhibits strong drought resistance, the strength of which is ranked as follows: Guangxi Leye > Yunnan Xinping > Guangxi Pingxiang [23]. Therefore, *A. odoratissima* is applied in the construction of large-scale forestry projects, for example, for the precise improvement of Guangxi’s forest quality, the national reserve forest, and the adjustment of tree species structure. However, the molecular mechanism of *A. odoratissima* seedlings’ response to drought stress is still unknown. In this study, the leaf morphology and structure, physicochemical characteristics, gene expression, and metabolite accumulation of *A. odoratissima* under drought stress were investigated, and the molecular mechanism of *A. odoratissima* seedlings’ response to drought stress was revealed. These findings provide a theoretical basis for the genetic breeding of *A. odoratissima*.

## 2. Results

### 2.1. Structural and Physiological Changes in A. odoratissima Seedlings under Drought Stress

**Figure 1:**
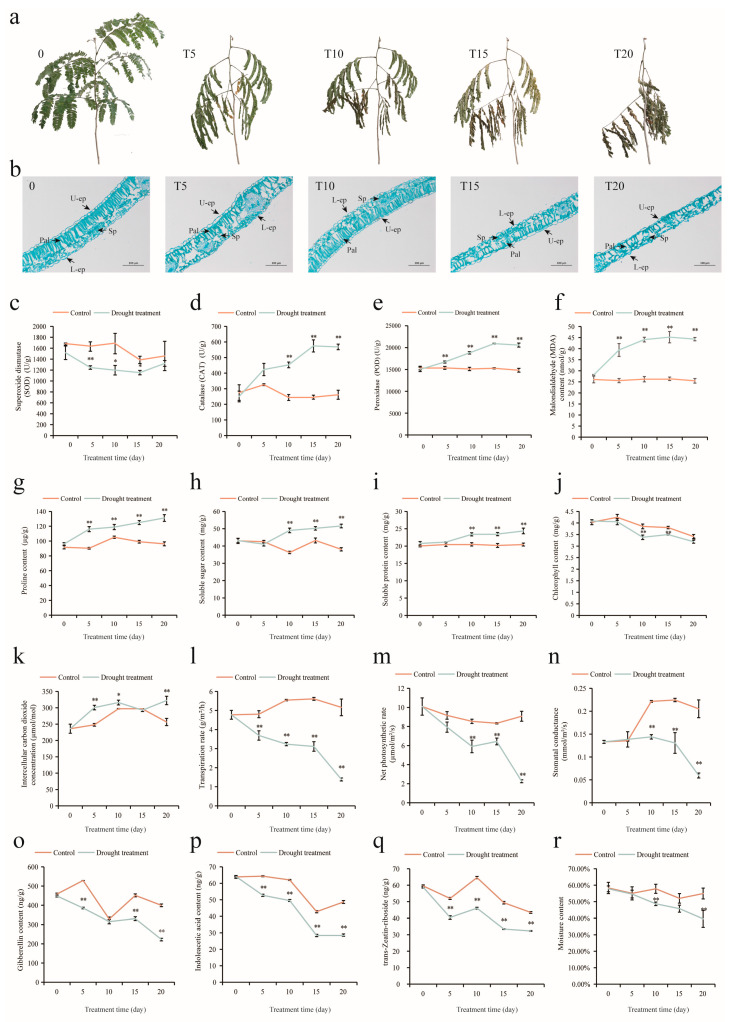
Structural and physiological biochemical changes in leaves of *A. odoratissima* seedlings under drought stress. (**a**) Changes in leaf shape on Days 0, 5, 10, 15, and 20. (**b**) Cross-sections of leaves (×100) (U-ep = upper epidermis, Pal = palisade, L-ep = lower epidermis). (**c**) Changes in SOD activity. (**d**) Changes in CAT activity in the leaves of *A. odoratissima* under drought stress. (**e**) Changes in POD activity. (**f**) Changes in MDA content. (**g**) Changes in proline content. (**h**) Changes in soluble sugar content. (**i**) Changes in soluble protein. (**j**) Changes in chlorophyll content. (**k**) Changes in intercellular carbon dioxide concentration. (**l**) Changes in transpiration rate. (**m**) Changes in net photosynthetic rate. (**n**) Changes in stomatal conductance. (**o**) Changes in gibberellin content. (**p**) Changes in indoleacetic acid content. (**q**) Changes in trans-Zeatin-riboside. (**r**) Changes in moisture content. *: significant difference between control and treatment. **: highly significant difference between control and treatment. T5, T10, T15, and T20: Days 5, 10, 15, and 20 of drought treatment.

With the gradual extension of drought stress treatment, the color of the *A. odoratissima* seedling leaf blades gradually changed from green to yellow, and the compound petioles gently drooped (Figure 1a). At the same time, the upper epidermis of the leaf blades thinned, the spongy tissues shortened and dispersed, the cell gaps widened, the main veins became smaller and fewer in number, and the xylem of the seedlings shortened, indicating that drought stress influenced the structure of the *A. odoratissima* seedlings (Figure 1b and Table S1).

Compared with the control groups, the chlorophyll content in the leaves of the experimental samples gradually decreased with the duration of drought stress, and it presented a significant difference from that in the control groups on the 10th and 15th days (Figure 1j). Meanwhile, the intercellular CO_2_ concentration (Ci) in the leaves significantly increased on the 5th and 20th days, while transpiration rate (Tr) gradually decreased with the increase in the duration of drought stress and exhibited notable differences from that of the control groups in different periods (Figure 1k,l). Under the same conditions, net photosynthetic rate (Pn) also decreased and showed obvious differences from that in the control groups on the 10th, 15th, and 20th days (Figure 1m). Furthermore, stomatal conductance (Gs) began to decrease on the 10th day under the drought stress treatment, and it was significantly lower than that in the control groups (Figure 1n).

The free proline, soluble sugar, and soluble protein contents in the leaves significantly increased with the duration of the drought stress treatment, and the free proline content was notably higher than that in the control groups during the corresponding periods (Figure 1g–i). Under the same conditions, the superoxide dismutase (SOD) activities in the leaves decreased, but the malondialdehyde (MDA) content increased, as well as the peroxidase (POD) and catalase (CAT) activities, compared with those in the control groups (Figure 1c–f). Meanwhile, the gibberellin (GA), indoleacetic acid (IAA), and zeatin riboside (ZR) contents in the leaves decreased compared with those in the control groups (Figure 1o–q). Moreover, the moisture content in the leaves of the *A. odoratissima* seedlings gradually decreased, with the difference from the control group becoming significant from the 10th day onward, indicating that drought stress affected the physiological properties of the leaves of the *A. odoratissima* seedlings (Figure 1r).

### 2.2. Transcriptome Analysis of A. odoratissima Seedlings under Drought Stress

**Figure 2:**
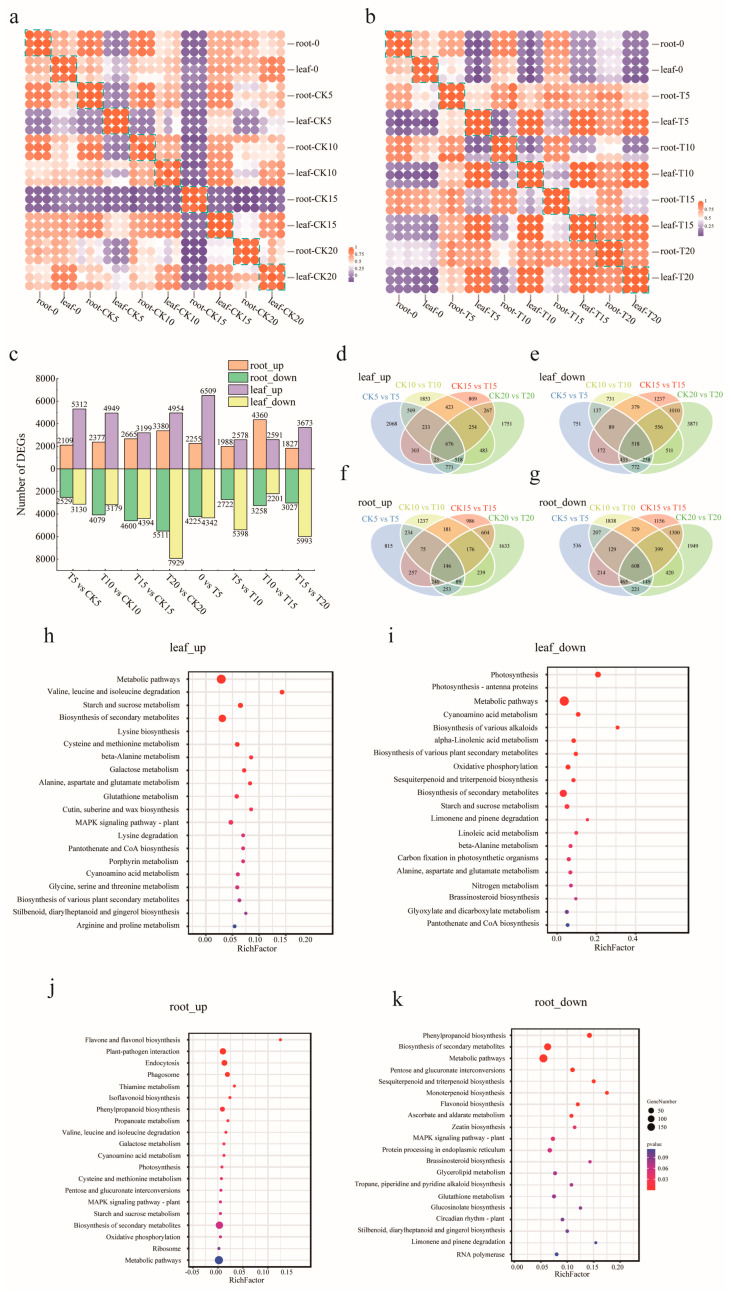
Analysis of transcriptional differences between the leaves and roots of *A. odoratissima* under drought stress. (**a**) Transcriptome correlation analysis under control. (**b**) Transcriptome correlation analysis under drought stress. (**c**) Changes in DEGs in the leaves and roots of *A. odoratissima* under drought stress. Up represents upregulated genes, and down represents downregulated genes. (**d**–**g**) Venn analysis ((**d**) leaf upregulated differential genes; (**e**) leaf downregulated differential genes; (**f**) root upregulated differential genes; (**g**) root downregulated differential genes). (**h**–**k**) KEGG pathways of differentially expressed genes ((**h**) leaf upregulated differential genes; (**i**) leaf downregulated differential genes; (**j**) root upregulated differential genes; (**k**) root downregulated differential genes). CK5, CK10, CK15, and CK20: Days 5, 10, 15, and 20 in the control groups. T5, T10, T15, and T20: Days 5, 10, 15, and 20 of drought treatment groups.

The RNA of the leaf and root samples was extracted and assessed, and transcriptome sequencing was subsequently performed. Over 6.6 Gb of clean data were generated for each sample, and Q20 ranged from approximately 96.33% to 98.31% (Table S2). The clean reads from the transcriptome sequencing were aligned with the reference genome of *A. odoratissima*, and the FPKM values were calculated. A correlation analysis of these FPKM values revealed a consistency of over 90% among the replicates for each sample, demonstrating the high reproducibility and reliability of the generated transcriptomic data (Figure 2a,b).

Within the groups of CK5 vs. T5, CK10 vs. T10, CK15 vs. T15, and CK20 vs. T20, a total of 5312, 4949, 3199, and 4954 differentially expressed genes (DEGs) in the leaves were upregulated, and a total of 3130, 3179, 4394, and 7929 DEGs in the leaves were downregulated (Figure 2c). A Venn analysis revealed that 676 DEGs were upregulated and that 518 DEGs were downregulated in the leaves in the four comparison groups (Figure 2d,e). The upregulated DEGs in the leaves were mainly annotated in the pathways of metabolism; the degradation of valine, leucine, and isoleucine; starch and sucrose metabolism; secondary metabolite biosynthesis; and lysine biosynthesis (Figure 2h). The upregulated DEGs were mainly involved in the processes of quercetin O-glucoside metabolism, kaempferol O-glucoside metabolism, beta-glucoside metabolism, s-glycoside catabolism, and glucosinolate catabolism (Figure S1). Conversely, the downregulated DEGs were annotated in the pathways of photosynthesis, photosynthetic antenna proteins, metabolism, cyanogenic glycoside metabolism, and the biosynthesis of various alkaloids (Figure 2i). A GO enrichment analysis showed that the downregulated DEGs were related to the photosynthesis, photoreaction, light harvesting by photosystem I, precursor metabolite, energy generation, and light-harvesting pathways (Figure S2). These results indicate that drought stress regulated the metabolic processes in the leaves of the *A. odoratissima* seedlings. 

Within the CK5 vs. T5, CK10 vs. T10, CK15 vs. T15, and CK20 vs. T20 groups, a total of 2190, 2377, 2665, and 3380 DEGs in the roots were upregulated, and a total of 2529, 4079, 4600, and 5511 DEGs in the roots were downregulated (Figure 2c). A Venn analysis of the DEGs revealed that 146 DEGs were upregulated and that 608 DEGs were downregulated in the roots in the four comparison groups at all four time points (Figure 2f,g). The upregulated DEGs in the roots were mainly annotated in the pathways of flavone and flavonol biosynthesis, plant–pathogen interactions, endocytosis, phagosomes, and isoflavonoid biosynthesis (Figure 2j). The upregulated DEGs were mainly involved in the processes of pollen maturation, the regulation of photosynthesis and photoreaction, flower organ development, ovule identity determination, and azole or thiazole biosynthesis (Figure S3). Conversely, the downregulated DEGs were annotated in the pathways of phenylpropanoid biosynthesis, secondary metabolite biosynthesis, metabolism, MAPK signaling, and thiamine metabolism (Figure 2k). A GO enrichment analysis showed that the downregulated DEGs were related to syncytium formation, cell wall modification for multidimensional cell growth, the negative regulation of cellular aging, and the regulation of cation channel activity (Figure S4). These results indicate that drought stress regulated the metabolic processes in the roots of the *A. odoratissima* seedlings. 

### 2.3. Metabolome Analysis of A. odoratissima Seedlings under Drought Stress

**Figure 3:**
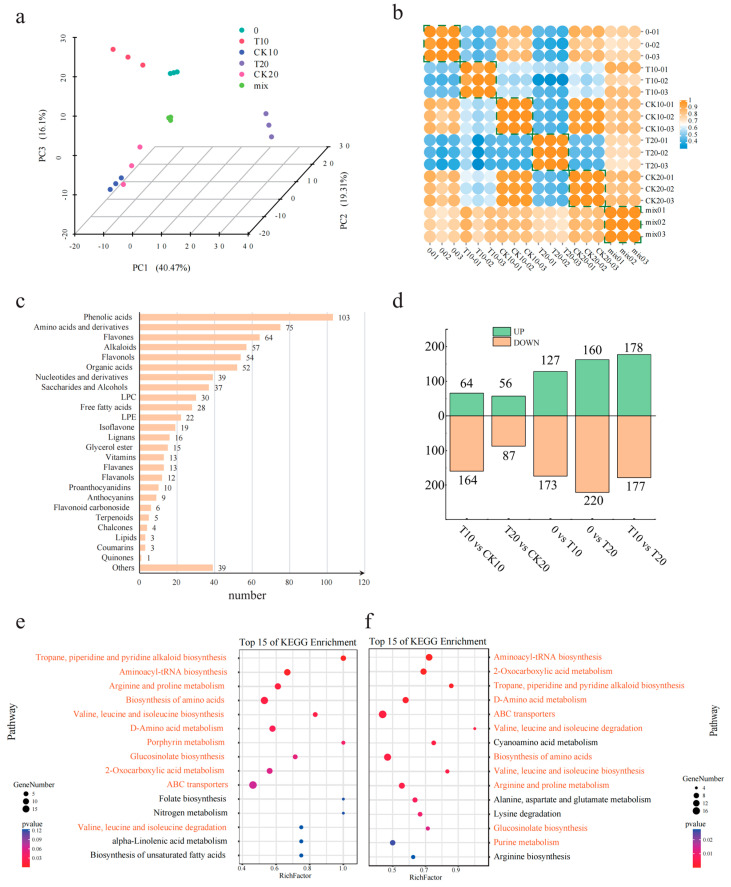
Differential analysis of metabolites in leaves of *A. odoratissima* under drought stress. (**a**) PCA of differential metabolites. (**b**) Correlation analysis of differential metabolites. (**c**) Differential metabolites in leaves. (**d**) Characterization of temporal changes in differential metabolites in leaves. (**e**) KEGG pathways of differential metabolites of *A. odoratissima* after 10 days of drought stress. (**f**) KEGG pathways of differential metabolites of *A. odoratissima* after 20 days of drought stress. Orange font indicates the same KEGG pathways of differential metabolites in both after 10 and 20 days.

The metabolites in the leaves of the *A. odoratissima* seedlings under drought stress were determined using non-targeted metabolome technology after 0, 10, and 20 d. A total of 729 metabolites grouped into 25 categories were identified, with the top 3 categories being phenolic acids (103 species, 14.03%), amino acids and their derivatives (75 species, 10.22%), and flavones (64 species, 8.72%) (Figure 3c). A PCA showed that PC1 accounted for 40.47%, PC2 accounted for 19.31%, and PC3 accounted for 16.10%, and three replicates of each of the five groups and quality controls revealed a high degree of correlation approaching 1 (Figure 3a,b). The results of both the PCA and correlation analysis indicated strong consistency among the three replicates of each of the five metabolic profile samples, enabling the five samples to be effectively distinguished and providing robust data support for subsequent analyses.

Differential accumulation metabolites (DAMs) were selected based on a standard fold change of ≥1.5 or ≤0.67 and a VIP ≥ 1. Within the CK10 vs. T10 group, a total of 228 DAMs were identified, among which 64 metabolites were upregulated, and 164 metabolites were downregulated. These were mainly enriched in tropane, piperidine, and pyridine alkaloid biosynthesis; aminoacyl-tRNA biosynthesis; arginine and proline metabolism; amino acid biosynthesis; and valine, leucine, and isoleucine biosynthesis (Figure 3e). Within the CK20 vs. T20 group, the treatment group had 143 DAMs, among which 56 metabolites were upregulated and 87 metabolites were downregulated (Figure 3d). These were mainly annotated in aminoacyl-tRNA biosynthesis; 2-oxocarboxylic acid metabolism; tropane, piperidine, and pyridine alkaloid biosynthesis; D-amino acid metabolism; and ABC transporters (Figure 3f).

### 2.4. Integrated Transcriptome and Metabolome Analysis of A. odoratissima Seedlings under Drought Stress

**Figure 4:**
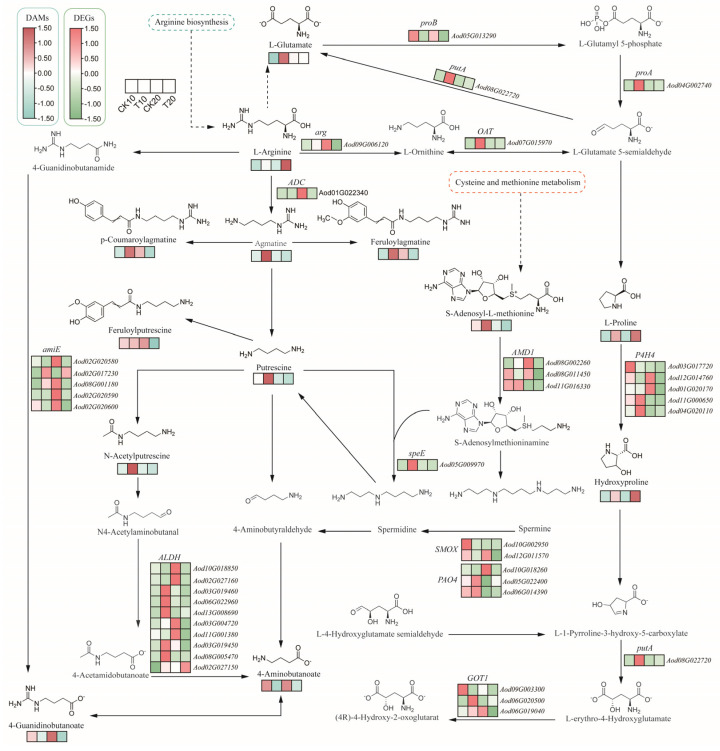
The arginine and proline metabolic pathways of *A. odoratissima* under drought stress. The colors indicate significances shown in a color scale. The solid lines with arrows indicate directions of the processes. Dashed lines represent the two-step and multi-step reactions.

Within the arginine and proline metabolic pathways, a total of 38 DEGs and 13 DAMs were identified. The 38 DEGs were *AoproB* (*Aod05G013290*) encoding glutamate 5-kinase, *AoproA* (*Aod04G002740*) encoding glutamate-5-semialdehyde dehydrogenase, *AoputA* (*Aod08G022720*) encoding 1-pyrroline-5- carboxylate dehydrogenase, *AoP4HA* (*Aod03G017720*/*12G014760*/*01G020170*/*11G000650*/*04G020110*) encoding prolyl 4-hydroxylase, *AoOAT* (*Aod07G015970*) encoding ornithine--oxo-acid transaminase, *Aoarg* (*Aod09G006120*) encoding arginase, *AoADC* (*Aod01G022340*) encoding arginine decarboxylase, *AoGOT1* (*Aod09G003300*/*06G020500*/*06G019040*) encoding aspartate aminotransferase, *AoAMD1* (*Aod08G002260*/*08G011450*/*11G016330*) encoding S-adenosylmethionine decarboxylase, *AoSMOX* (*Aod10G002950*/*12G011570*) encoding spermine oxidase, *AoPAO4* (*Aod10G018260*/*05G022400*/*06G014390*) encoding polyamine oxidase, *AospeE* (*Aod05G009970*) encoding spermidine synthase, *AoALDH* (*Aod10G018850*/*02G027160*/*03G019460*/*06G022960*/*13G008690*/*03G004720*/*11G001380*/*03G019450*/*08G005470*/*02G027150*) encoding aldehyde dehydrogenase, and *AoamiE* (*Aod02G020580*/*02G017230*/*08G001180*/*02G020590*/*02G020600*) encoding amidase. Drought stress upregulated the expression of the *AoproA* and *AoOAT* genes and increased the levels of arginine (kz000360) and proline (kz000318) (Figure 4). These results show that drought stress promoted proline biosynthesis by improving the expression levels of crucial genes in its metabolic pathway in the leaves of the *A. odoratissima* seedlings. 

**Figure 5:**
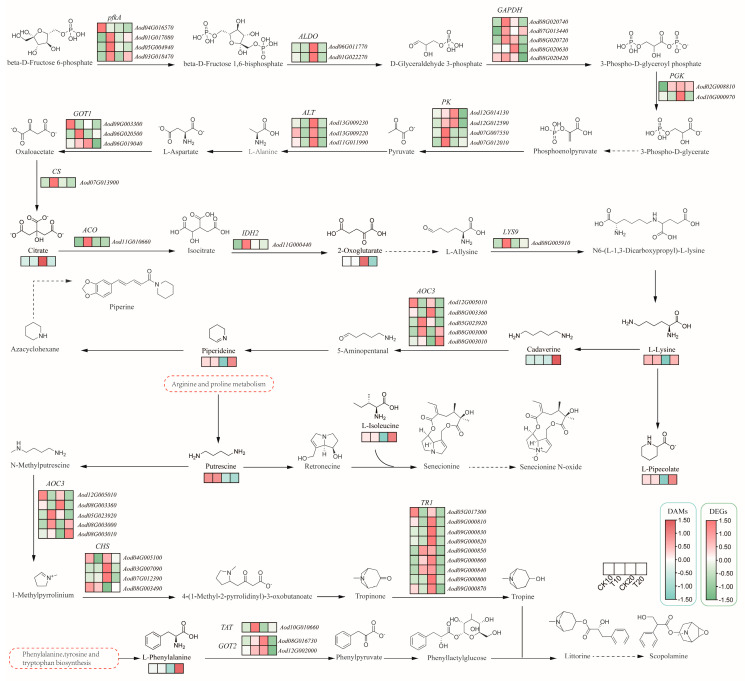
The tropane, piperidine, and pyridine alkaloid biosynthetic pathways of *A. odoratissima* under drought stress.

Within the tropane, piperidine, and pyridine alkaloid biosynthetic pathways, a total of 48 DEGs and 9 DAMs were identified. The 48 DEGs were *AopfkA* (*Aod04G016570*/*01G017080*/*05G004940*/*03G018470*) encoding 6-phosphofructokinase 1, *AoALDO* (*Aod06G011770*/*01G022270*) encoding fructose-bisphosphate aldolase, *AoGAPDH* (*Aod08G020740*/*07G013440*/*08G020720*/*08G020630*/*08G020420*) encoding glyceraldehyde 3-phosphate dehydrogenase (phosphorylating), *AoPGK* (*Aod02G008810*/*10G000970*) encoding phosphoglycerate kinase, *AoPK* (*Aod12G014130*/*12G012590*/*07G007550*/*07G012010*) encoding pyruvate kinase, *AoALT* (*Aod13G009230*/*13G009220*/*11G011990*) encoding alanine transaminase, *AoGOT1* (*Aod09G003300*/*06G020500*/*06G019040*) encoding aspartate aminotransferase, cytoplasmic, *AoCS* (*Aod07G013900*) encoding citrate synthase, *AoACO* (*Aod11G010660*) encoding aconitate hydratase, *AoIDH2* (*Aod11G000440*) encoding isocitrate dehydrogenase, *AoLYS9* (*Aod08G005910*) encoding saccharopine dehydrogenase, *AoAOC3* (*Aod12G005010*/*08G003360*/*05G023920*/*08G003000*/*08G003010*) encoding primary-amine oxidase, *AoCHS* (*Aod04G005100*/*03G007090*/*07G012390*/*08G003490*) encoding chalcone synthase, *AoTR1* (*Aod05G017300*/*09G000810*/*09G000830*/*09G000820*/*09G000850*/*09G000860*/*09G000840*/*09G000800*/*09G000870*) encoding tropinone reductase I, *AoTAT* (*Aod10G010660*) encoding tyrosine aminotransferase, and *AoGOT2* (*Aod08G016730*/*12G002000*) encoding aspartate aminotransferase, mitochondrial. Drought stress upregulated the expression of the *AopfkA* (*Aod01G017080*/*03G018470*) and *AoGAPDH* (*Aod07G013440*) genes and increased the content of lysine (kz000339), a precursor of tropane, piperine, and pyridine alkaloid biosynthesis (Figure 5), whereas drought stress upregulated *AoAOC3* (*Aod08G003000*/*08G003010*) gene expression and increased the content of lysine (kz000339), the precursor of cadaverine (kz000041) and piperidine (kz000229) in the piperine synthetic pathway. These results indicate that drought stress promoted the biosynthesis of tropane, piperidine, and pyridine alkaloids by increasing the expression levels of key genes in the leaves of the *A. odoratissima* seedlings.

### 2.5. Weighted Gene Co-Expression Network Analysis of A. odoratissima under Drought Stress

Data on soluble sugar, soluble protein, GA, Pn, proline, POD, and CAT were used as trait files, and, by using all 54 transcriptome datasets (Table S2), 35 modules were ultimately constructed using the weighted gene co-expression network analysis (WGCNA) method. Among these 35 modules, the blue and cyan modules exhibited significant positive correlations with GA and Pn, and they exhibited significant negative correlations with proline, soluble protein, soluble sugar, CAT, and POD (Figure S5). 

The blue module contained 3256 genes, which were mainly annotated in the pathways of photosynthesis antenna proteins; carbon fixation in photosynthetic organisms; photosynthesis; carbon metabolism; glyoxylate and dicarboxylate metabolism; glycine, serine, and threonine metabolism; and the citrate cycle (TCA cycle) (Figure S6). A GO enrichment analysis showed that these genes were enriched in the processes of photosynthesis, photosynthesis photoreaction, photosynthesis light harvesting, photosynthesis light harvesting in photosystem I, RNA modification, metabolism, and nucleic acid phosphodiester bond hydrolysis (Figure S7). In the cyan module, a total of 571 genes were identified in the KEGG database, which were primarily annotated in the pathways of glycosaminoglycan degradation; protein processing in the endoplasmic reticulum; carbon metabolism; sulfur metabolism; fatty acid metabolism; porphyrin metabolism; valine, leucine, and isoleucine degradation; and the biosynthesis of unsaturated fatty acids (Figure S8). A GO enrichment analysis showed that these genes were highlighted in the processes of plant-type secondary cell wall biogenesis, plant-type cell wall biogenesis, cell wall biogenesis, polysaccharide biosynthesis, single-organism carbohydrate metabolism, carbohydrate biosynthesis, carbohydrate metabolism, polysaccharide metabolism, and cellular polysaccharide biosynthesis (Figure S9). 

**Figure 6:**
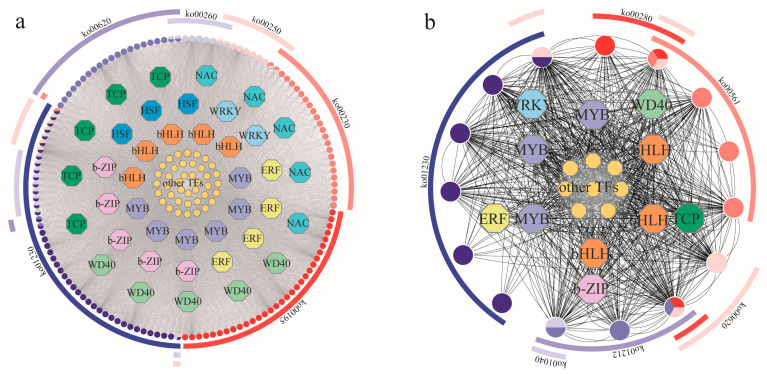
WGCNA of physiological indicators and transcriptional differences in *A. odoratissima* under drought stress. (**a**) Co-expression network of blue module and drought-stress-related genes (ko00620: pyruvate metabolism; ko00260: threonine metabolism; ko00250: glutamate metabolism; ko00230: purine metabolism; ko00195: photosynthesis; ko01230: amino acid biosynthesis). (**b**) Co-expression network of cyan module and drought-stress-related genes (ko01230: amino acid biosynthesis; ko00280: isoleucine degradation; ko00561: glycerolipid metabolism; ko00620: pyruvate metabolism; ko01212: fatty acid metabolism; ko01040: unsaturated fatty acids).

Based on the criteria |Gene Significance (GS)| > 0.85 or |Module Membership (MM)| > 0.9, the hub genes in the blue and cyan modules were screened and identified. In the blue module, *MYB* (6), *bHLH* (7), *b-ZIP* (5), *TCP* (5), *WRKY* (2), *NAC* (5), *WD40* (5), *ERF* (4), *HSF* (3), and several other transcription factors were obtained, and they were mainly annotated in the pathways of photosynthesis (ko00195); purine metabolism (ko00230); alanine, aspartate, and glutamate metabolism (ko00250); glycine, serine, and threonine metabolism (ko00260); amino acid biosynthesis (ko01230); and pyruvate metabolism (ko00620) (Figure 6a). Within the cyan module, *MYB* (3), *bHLH* (2), *b-ZIP* (1), *TCP* (1), *WRKY* (1), *WD40* (1), *ERF* (1), and multiple other transcription factors were identified, and they were enriched in the processes of fatty acid metabolism (ko01212); valine, leucine, and isoleucine degradation (ko00280); the biosynthesis of unsaturated fatty acids (ko01040); glycerolipid metabolism (ko00561); amino acid biosynthesis (ko01230); and pyruvate metabolism (ko00620) (Figure 6b). In order to further verify the effects of drought stress on the gene expression levels of the *A. odoratissima* seedlings, 16 key functional genes were randomly selected for qRT-PCR verification (Figure S10). The results show that the expression levels of the above 16 genes were basically consistent with the RNA-seq results, indicating that the sequencing results are reliable.

## 3. Discussion

### 3.1. Drought Stress Inhibits the Growth of A. odoratissima Seedlings

Water serves as a solvent and regulates the physiological metabolic reactions in cells, eventually affecting the growth and development of plants [24]. Drought stress is caused by water loss in soil and, in turn, influences the pigment content, photosynthetic efficiency, and ultrastructure in plants [25,26]. In the present study, the moisture content in the leaves of *A. odoratissima* seedlings gradually decreased with the increase in drought duration, and significant differences emerged on the 10th day, indicating that drought reduced the moisture content in the leaves of the *A. odoratissima* seedlings. Under the same conditions, the leaf mesophyll structures and leaf vein thickness of the *A. odoratissima* seedlings decreased compared with those of the control group, suggesting that drought stress impedes the water flow in cells, thereby enhancing the adaptability of *A. odoratissima* seedlings [27]. At the same time, leaf development was associated with the intracellular hormone content. In the drought stress treatment, the zeatin riboside (ZR) content in the leaves decreased and then increased, but the indoleacetic acid (IAA) and gibberellin (GA) contents in the leaves gradually decreased with prolonged drought stress, indicating that drought stress inhibited leaf development by reducing the endogenous hormone contents, thereby delaying the growth and improving the drought tolerance of the *A. odoratissima* seedlings [28]. 

Chlorophyll is the main carrier of photosynthesis in leaves, and its content determines the photosynthetic capacity and growth rate of plants, but its biosynthesis and accumulation are affected by drought stress [29]. In this experiment, drought stress reduced the total chlorophyll content while concurrently decreasing transpiration rate (Tr) and net photosynthetic rate (Pn) but increased the intercellular CO_2_ concentration (Ci) in the leaves, suggesting that drought stress reduced the photosynthetic capacity by decreasing the total chlorophyll content in the leaves of the *A. odoratissima* seedlings. The decrease in the total chlorophyll content reduced the light capture and utilization ability, subsequently reduced the photosynthetic capacity, and eventually inhibited growth and tissue development, thereby improving the tolerance of the *A. odoratissima* seedlings [30]. However, drought stress induced stomatal closure, which controls gas exchange and water loss, to increase Tr and Pn, ultimately delaying the growth and tissue development of the leaves of the *A. odoratissima* seedlings [31]. In order to alleviate the adverse effects of drought stress, leaves can reduce transpiration. This subsequently enhances the accumulation of Ci, which inhibits photosynthesis at high concentrations and, in turn, reduces the photosynthetic capacity and growth of *A. odoratissima* seedlings to improve adaptability [32].

### 3.2. Drought Stress Improves the Antioxidant Capacity of A. odoratissima Seedlings

Drought stress rapidly induces ROS production, promotes lipid peroxidation and malondialdehyde (MDA) formation, destroys the integrity of cell membranes, and alters the morphology and structure of tissues and organs, ultimately inhibiting the growth, yield, and quality of plants [33,34]. The previous result showed that drought stress induced MDA formation and promoted the biosynthesis and accumulation of soluble substances, thereby regulating the osmotic pressure and water potential in cells and thus enhancing the tolerance of plants [35]. In the present study, drought stress increased the MDA content in the leaves, and the proline, soluble sugar, and soluble protein contents in the leaves also increased with an increasing duration of drought stress, indicating that drought stress promoted lipid peroxidation and the accumulation of soluble substances in the leaves of the *A. odoratissima* seedlings. Proline, as an important amino acid, has the crucial functions of regulating osmosis, reducing acidity, enhancing the redox capacity, and maintaining the structural stability of cells, and plants can promote its synthesis and accumulation to improve their drought resistance [36]. Soluble sugar and protein are mainly derived from photosynthetic products and the degradation of biological macromolecules, which play roles in osmotic regulation and water potential, as well as providing energy and a substrate for substance biosynthesis in cells, thereby regulating growth and adaptability to drought stress [37,38]. Therefore, *A. odoratissima* seedlings demonstrated enhanced drought tolerance as a result of the promoted accumulation of soluble substance in their leaves.

Drought stress not only influences the content of soluble substances but also regulates the activity of the antioxidase system in plants. The main antioxidases are superoxide dismutase (SOD), peroxidase (POD), and catalase (CAT), which can increase adaptability by clearing the ROS in plants [39]. In this study, drought stress decreased the SOD activity but increased the POD and CAT activities in the leaves, indicating that the *A. odoratissima* seedlings demonstrated improved drought resistance as a result of enhanced POD and CAT activities in their leaves. SOD catalyzes the formation of hydrogen peroxide by the superoxide anion, but the decrease in the SOD activity in the leaves indicates that the superoxide anion is not the predominant ROS type induced by drought stress [40]. POD and CAT mainly remove the hydrogen peroxide in cells; thus, an increase in these enzymes’ activities can mitigate the damage to and improve the drought tolerance of plants [41]. These results indicate that the *A. odoratissima* seedlings demonstrated improved adaptability and drought resistance as a result of enhanced POD and CAT activities in their leaves.

### 3.3. Drought Stress Promotes the Accumulation of Amino Acids and Alkaloids

Drought stress regulates gene expression and metabolic processes, which, in turn, affect plant growth [42]. In the present study, a total of 676 differentially expressed genes (DEGs) were upregulated and 518 DEGs were downregulated in leaves of *A. odoratissima* seedlings. The upregulated DEGs were mainly enriched in the pathways of metabolism; valine, leucine, and isoleucine degradation; starch and sucrose metabolism; secondary metabolite biosynthesis; and lysine biosynthesis. In contrast, the downregulated DEGs were annotated in the pathways of photosynthesis, photosynthesis–haptoglobin, metabolism, cyanogenic glycoside metabolism, and alkaloid biosynthesis. This suggests that drought stress affects physiological and metabolic processes by regulating the gene expression in the leaves of *A. odoratissima* seedlings. In the drought stress treatment, within the CK10 vs. T10 group, a total of 228 differential accumulation metabolites (DAMs) were identified, among which 64 metabolites were upregulated, and 164 metabolites were downregulated. Within the CK20 vs. T20 group, the treatment group had 143 DAMs, among which 56 metabolites were upregulated, and 87 metabolites were downregulated. The top five bioprocesses of the upregulated DAMs were metabolic pathways; valine, leucine, and isoleucine degradation; starch and sucrose metabolism; secondary metabolite biosynthesis; and lysine biosynthesis. Furthermore, the downregulated DAMs were primarily enriched in photosynthesis, photosynthetic antenna proteins, metabolic pathways, cyanogenic amino acid metabolism, and the biosynthesis of various alkaloids. This indicates that drought stress had different effects on the physicochemical and metabolic processes in the leaves of the *A. odoratissima* seedlings. Amino acids play a variety of roles in regulating plant tolerance to abiotic stress, including roles as osmotic regulators, ROS scavengers, and precursors of energy-related metabolites [43,44]. In the amino acid metabolism pathway, drought stress enhanced the relative expression levels of the *AoproA* and *AoOAT* genes, and it increased the arginine (kz000360) and proline (kz000318) contents in the leaves, showing that drought stress promotes proline biosynthesis by increasing the relative expression levels of crucial genes in the leaves of *A. odoratissima* seedlings. Proline can polymerize with some compounds in the plant cytosol to form hydrophilic colloid-like compounds, thereby reducing the osmotic pressure and water potential in cells while maintaining the structural integrity of the cell organism, ultimately improving the drought resistance of *A. odoratissima* seedlings [45,46]. 

Drought stress promotes the biosynthesis and accumulation of secondary metabolites in plants, among which alkaloids are important. Alkaloids play crucial roles in the growth and drought resistance of plants [47]; they can interplay with molecules, maintain the structural stability and activity of macromolecules, and scavenge the ROS induced in cells to alleviate membrane damage, eventually improving the adaptability and drought resistance of plants [48,49,50]. In this study, drought stress increased the relative expression level of *AoAOC3* (*Aod08G003000*/*08G003010*, *AMINE OXIDASE COPPER CONTAINING 3*) and the contents of cadaverine (kz000041), piperidine (kz000229), and the precursor lysine (kz000339) in the piperine synthesis pathway, suggesting that drought stress promoted the biosynthesis and accumulation of alkaloids by enhancing the relative expression level of the *AoAOC3* gene in the leaves of the *A. odoratissima* seedlings. Cadaverine and piperidine belong to the alkaloid family. Their synthesis and accumulation could increase their antioxidant capacity, and, together with antioxidases, they could reduce ROS-mediated oxidative damage, in turn, improving the drought resistance of the *A. odoratissima* seedlings. 

### 3.4. Identification of Key Transcription Factors in Response to Drought Stress

The key transcription factors in the response to drought stress in the leaves of the *A. odoratissima* seedlings were identified using WGCNA technology [51]. Some crucial genes encoding transcription factors were identified, including the *MYB*, *bHLH*, *b-ZIP*, *TCP*, and *WRKY* families, of which the *MYB* and *bHLH* families accounted for the highest number of genes. Previous results showed that the *MYB* and *bHLH* family genes regulated the biosynthesis and accumulation of flavonoids and alkaloids, which could remove the ROS in and mitigate their damage to cells, subsequently enhancing the adaptability and tolerance of plants under abiotic stress [52,53]. Therefore, we speculate that drought stress improves the resistance of *A. odoratissima* seedlings by regulating the expression of key transcription factors in the leaves. However, the biological functions of these key genes in *A. odoratissima* seedlings’ response to drought stress remain unclear, and this will be the focus of our future research.

**Figure 7:**
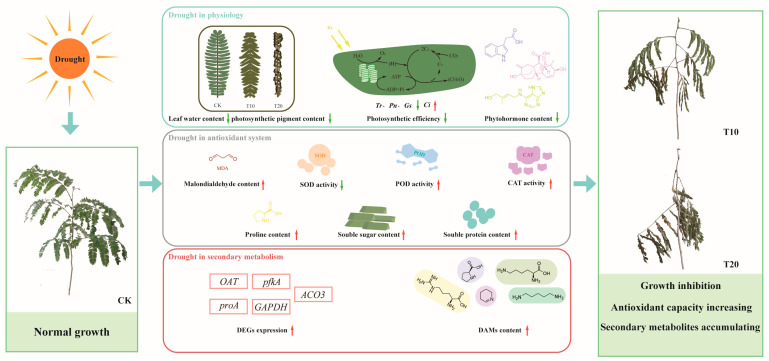
A conceptual model of the drought response mechanism in *A. odoratissima*. Red colour indicates increased content, enzyme activity or up-regulated gene expression, green colour indicates decreased content, enzyme activity or down-regulated gene expression.

Based on these results, a conceptual model of the molecular mechanism of the *A. odoratissima* seedlings’ response to drought stress was developed (Figure 7). Briefly, drought stress reduced the moisture content, total chlorophyll content, photosynthetic capacity, IAA and GA contents, SOD activity, and leaf mesophyll and vein thicknesses, but it increased the MDA content, POD and CAT activities, and proline, soluble sugar, and soluble protein contents, subsequently improving the adaptability and resistance of the *A. odoratissima* seedlings. However, drought stress enhanced the relative expression levels of the *AoproA*, *AoOAT*, *AopfkA*, *AoGAPDH*, and *AoAOC3* genes, and it promoted the biosynthesis and accumulation of arginine, proline, cadaverine, piperidine, and lysine metabolites, thereby strengthening the drought tolerance of the *A. odoratissima* seedlings.

## 4. Materials and Methods

### 4.1. Design of Experiments

#### 4.1.1. Drought-Stressed Environment

A drought stress test was conducted in a greenhouse to avoid the influence of possible rainfall on the test process, while the air temperature and humidity in the greenhouse were kept essentially the same as the outdoor environment via artificial adjustment.

#### 4.1.2. Pre-Experiment

In July 2021, five *A. odoratissima* seedlings were placed in the greenhouse, and artificial watering was stopped so that the growth and phenotypic changes in the seedlings under natural drought conditions could be observed and recorded every day. The pre-experiment results showed that all of the *A. odoratissima* seedlings died, the leaves dried and withered, and growth did not resume following re-watering after 22 d of natural drought.

#### 4.1.3. Drought Stress Experiment

Sample trees were collected from a natural forest of *A. odoratissima* nilotica in the dry–hot valley area of the Nanpanjiang River (106°17′1″ E, 24°51′47″ N), with a straight-line distance of 7 km from the Nanpanjiang River to the sample trees. Young stem segments of the sample wood were used as propagation materials, and an asexual group of seedlings was cultivated and used for the drought stress test. 

The drought experiment was located at the experimental base of the Guangxi Forestry Research Institute, under natural light conditions, with an average temperature of 21.6 °C. The seedling container was a plastic pot with a width of 20 cm, a bottom diameter of 12 cm, and a height of 6.5 cm. The seedling substrate was made according to a yellow heart soil–coconut husk–cereal husk ratio of 5:3:2, and the weight of the substrate in each pot was determined to be 1.81 kg, which ensured the normal growth of the seedlings. The average seedling height was 80.3 cm, and the average diameter was 0.67 cm.

Prior to the drought stress test, the test seedlings received normal daily care to ensure normal growth. Based on the results of the pre-experiment, the duration of the drought stress test was set to 20 d, which was divided into four periods of 5 d, 10 d, 15 d, and 20 d. The test groups were divided into control groups (CK5, CK10, CK15, and CK20) and drought stress treatment groups (T5, T10, T15, and T20), with 60 seedlings in each group, divided into three biological replications, and sampling was carried out and related indices calculated at 0, 5, 10, 15, and 20 d. The drought stress test was terminated after 20 d.

### 4.2. Measurement of Physiological Indicators

#### 4.2.1. Total Chlorophyll Content in the Leaves

After sample collection, the total chlorophyll content in leaves of *A. odoratissima* seedlings was determined using the acetone–ethanol extraction method [54].

#### 4.2.2. Photosynthetic Parameters

Three *A. odoratissima* seedlings were randomly selected from the control and stress groups, and each seedling was assigned a number. The transpiration rate (Tr), net photosynthetic rate (Pn), intercellular CO_2_ concentration (Ci), and stomatal conductance (Gs) of the same-position leaves (counted from the top bud, either the third or fourth) of these numbered seedlings were then determined using an Li-6400 photosynthesis system produced in the United States. The water use efficiency (WUE) was calculated, and then the average value of each measured index was calculated. A red and blue light source (LI-6400-2B) was used, and the light intensity and the atmospheric CO_2_ concentration ranges were set to 1000 mol/m^2^/S and 450~500 µmol/mol, respectively. The measurements were performed within the temperature range of 30~33 °C at 8:30~11:30 in the morning every 5 days.

#### 4.2.3. Osmosis-Regulating Substances

The proline content in the samples was determined using the acid ninhydrin method. The concentration of soluble proteins was determined using the Coomassie brilliant blue method. The amount of soluble sugars was determined using the anthrone colorimetric method [55].

#### 4.2.4. Enzyme Activity

Malondialdehyde (MDA) levels were measured using the thiobarbituric acid method [56]. Peroxidase (POD) activity was measured using the guaiacol colorimetric method. Catalase (CAT) activity was measured using the ultraviolet absorption method [57]. Superoxide dismutase (SOD) activity was determined using the nitroblue tetrazolium photoreduction method [58].

#### 4.2.5. Relative Water Content in the Leaves

First, the leaves of the *A. odoratissima* seedlings collected from the control and stress groups were cut into small pieces, preheated, and then weighed on a balance. The weight at this point was denoted as W1. Afterward, the leaves were dried in an oven at 105 °C for 15 min, following which the temperature was reduced to 80 °C and maintained until a constant weight was reached. Finally, after cooling, the dry mass was weighed and recorded as W2. The relative water content (RWC) was then calculated using the following formula:

RWC = (W1 − W2)/W1 × 100%(1)

#### 4.2.6. Endogenous Hormones

The levels of gibberellin (GA), indoleacetic acid (IAA), and zeatin riboside (ZR) were determined using an enzyme-linked immunosorbent assay (ELISA) with the double-antibody sandwich method [59].

### 4.3. Transcriptome Analysis

The leaf and root tissues of the *A. odoratissima* seedlings under drought stress and those of the seedlings in the control group were collected after 0 d, 5 d, 10 d, 15 d, and 20 d. Three replicates were prepared for each sample. RNA was extracted from these tissue samples using an RNeasy Plant Mini Kit (Qiagen, Hilden, Germany). The extracted RNA was subjected to a quality evaluation using an electrophoresis system and an Agilent 2100 (Agilent Technologies, Santa Clara, CA, USA) and then used for the preparation of cDNA libraries that were later sequenced on an Illumina Novaseq 6000 platform (NovaSeq 6000, Illumina, San Diego, CA, USA) using 150 bp paired-end sequencing. The transcriptome data were evaluated using FastQC, version 0.11.5 (http://www.bioinformatics.babraham.ac.uk/projects/fastqc/ (accessed on 10 January 2023)), and low-quality reads were filtered out using Trimmomatic v0.33 software. High-quality reads were aligned to the reference genome of *A. odoratissima* using HISAT2, version 2.0.5 (http://ccb.jhu.edu/software/tophat/index.shtml (accessed on 3 March 2023)), software. The number of reads aligned to unique positions was calculated using StringTie (v1.3.3b) software [60], and the expression level of FPKM was calculated. The gene counts in each sample were normalized using DESeq2, version 1.16.1.t (http://bioconductor.org/packages/DESeq2/ (accessed on 13 April 2023)) (base mean value for expression estimation). The fold changes were calculated, and the differential significance of the reads was evaluated using negative binomial distribution (NB) testing. Finally, the differential protein-coding genes were selected based on the determined fold changes and the results of the differential significance testing using the criteria of *p*-value < 0.05 and fold change > 2. A Venn analysis was performed on the differentially expressed genes (DEGs) of different categories, and KEGG and GO enrichment analyses were performed on the shared DEGs. 

### 4.4. Metabolome Analysis

The leaf tissues of the *A. odoratissima* seedlings were collected from the drought stress and control groups after 0 d, 10 d, and 20 d. Three replicates were prepared for each sample. The leaves were freeze-dried, ground into powder, and subjected to 70% methanol extraction to obtain metabolites from the samples. A metabolome analysis was performed using a UPLC-MS/MS system. The chromatographic column used was a Waters ACQUITY UPLC HSS T3 C18 (1.8 µm, 2.1 mm * 100 mm). The mobile phase comprised ultrapure water with 0.1% formic acid (solvent A) and acetonitrile with 0.1% formic acid (solvent B). The elution gradient was as follows: 0 min, 95% A/5% B; 10.0 min, 5% A/95% B; 11.0 min, 5% A/95% B; 11.1 min, 95% A/5% B; 15.0 min, 95% A/5% B. The source temperature of the electrospray ionization equipment was set to 550 °C. The mass spectrum equipment voltage was set to [5500 V (positive), −4500 V (negative)]. The gas ion source I (GS I) was used at 55 psi, gas source II (GS II) was used at 60 psi, and curtain gas (CUR) was used at 25 psi. The collision-activated dissociation parameters were set to high. In the triple quadrupole mass spectrometer, each ion pair was scanned and detected based on the optimized declustering potential and collision energy. The mass spectrometry data were analyzed using Analyst 1.6.3 software. Qualitative and quantitative mass spectrometry analyses of the metabolites extracted from the samples were performed using the local metabolic database. A principal component analysis of the samples (including quality control samples) was then conducted for a preliminary assessment of the overall metabolic differences between the groups and the variability within each group. An orthogonal partial least squares discriminant analysis (OPLS-DA) was then performed on the metabolites to obtain the variable importance in projection for the OPLS-DA model. In addition, a univariate analysis was conducted to determine the differential metabolites based on the established criteria of fold change ≥ 1.5 or fold change ≤ 0.67 and VIP ≥ 0.1.

### 4.5. Integrated Transcriptome and Metabolome Analysis

An integrated transcriptome and metabolome analysis was performed to reveal the important metabolic pathways involved in the response of this species to drought stress, and the changes in the important metabolites and genes related to the key enzymes of these pathways were explored in detail. DEGs and DAMs in metabolic pathways such as arginine and proline and alkaloids in CK10 vs. T10 and CK20 vs. T20 were standardized and visualized by TBtools 1.098 and plotted using Adobe illustrator 2022 software.

### 4.6. WGCNA Co-Expression Network Construction

The expression data from the WGCNA of the leaf tissues of the *A. odoratissima* seedlings under drought stress were used to construct a co-expression network using the WGCNA package in R 4.3.3 software [51]. The data on the soluble sugar content, soluble protein content, gibberellin content, net photosynthesis rate, proline, POD, and CAT enzyme activity in the *A. odoratissima* seedlings under drought stress were imported as trait files. The results were visualized using Cytoscape (v3.7.1) software [61].

### 4.7. Statistical Analysis

In terms of statistical analyses, the data were collated using WPS Office 12.1.0.17827 software and SPSS 20.0 software for a one-way ANOVA, and the least significant difference (LSD) method was used for multiple comparisons, with significant differences defined at *p* < 0.05. All data are presented as the means ± standard errors of three replicates.

## 5. Conclusions

In summary, sustained drought stress decreased the moisture content, mesophyll and vein thicknesses, and photosynthetic capacity in the leaves of *A. odoratissima* seedlings, subsequently improving their adaptability. At the same time, drought stress increased the MDA, proline, soluble sugar, and protein contents, as well as the POD and CAT activities, in the leaves, thereby improving the drought resistance of the *A. odoratissima* seedlings. In drought treatment, a total of 1194 DEGs and 371 DAMs were identified. These were mainly annotated in the amino acid and alkaloid metabolism pathways, in which the arginine, proline, lysine, cadaverine, and piperidine metabolite contents were increased and the relative expression levels of the *AoproA*, *AoOAT*, *AopfkA*, *AoGAPDH*, and *AoAOC3* genes were upregulated. This indicates that drought stress promoted the biosynthesis and accumulation of amino acids and alkaloids by increasing the expression of key genes in metabolic pathways, suggesting that these predominant metabolites may improve the drought tolerance of *A. odoratissima* seedlings. Furthermore, some crucial transcription factors were identified using WGCNA technology. These results suggest that *A. odoratissima* seedlings respond to drought stress mainly by improving the capacities of the antioxidant system and secondary metabolism.

## Data Availability

The data used in this study will be shared on reasonable request to the corresponding author. The raw genome and transcriptome sequencing data of C. wilsoniana have been deposited in the Genome Sequence Archive (https://www.ncbi.nlm.nih.gov/ (accessed on 1 February 2024), accession no. PRJCA023416).

## Supplementary Materials

The following supporting information can be downloaded at https://www.mdpi.com/article/10.3390/plants13192732/s1, Figure S1: GO term enrichment of the genes with upregulated expression in the leaves of *A. odoratissima.* Figure S2: GO term enrichment of the genes with downregulated expression in the leaves of *A. odoratissima*. Figure S3: GO term enrichment of the genes with upregulated expression in roots of *A. odoratissima*. Figure S4: GO term enrichment of the genes with downregulated expression in roots of *A. odoratissima*. Figure S5: Correlation heat map of different modules and traits in the WGNNA. Figure S6: KEGG term enrichment of the genes in the blue module. Figure S7: GO term enrichment of the genes in the blue module. Figure S8: KEGG term enrichment of the genes in the cyan module. Figure S9: GO term enrichment of the genes in the cyan module. Figure S10: Validation of DEGs using qRT-PCR. Table S1: Effect of drought days on the leaf pulp of *A. odoratissima* seedlings. Table S2: Summary of transcriptome data.
