# Supplementary material for: Transcriptome and Metabolome Analyses Reveal the Molecular Mechanisms of Albizia odoratissima’s Response to Drought Stress
Source: Plants (Basel). 2024 Sep 29;13(19):2732. doi: 10.3390/plants13192732 (PMC11478484; doi:10.3390/plants13192732)

Figure S1 GO term enrichment of the genes with up-regulated expression in leaves of ***A. odoratissima***.

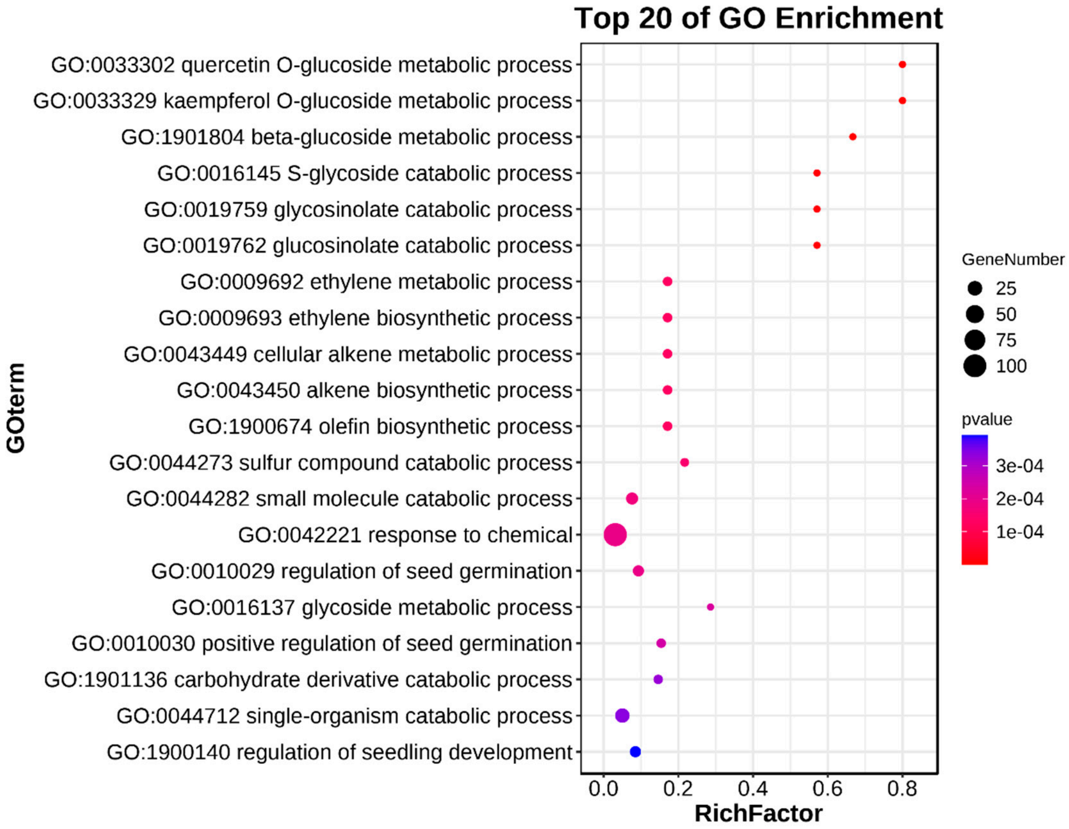

Figure S2 GO term enrichment of the genes with down-regulated expression in leaves of ***A. odoratissima***.

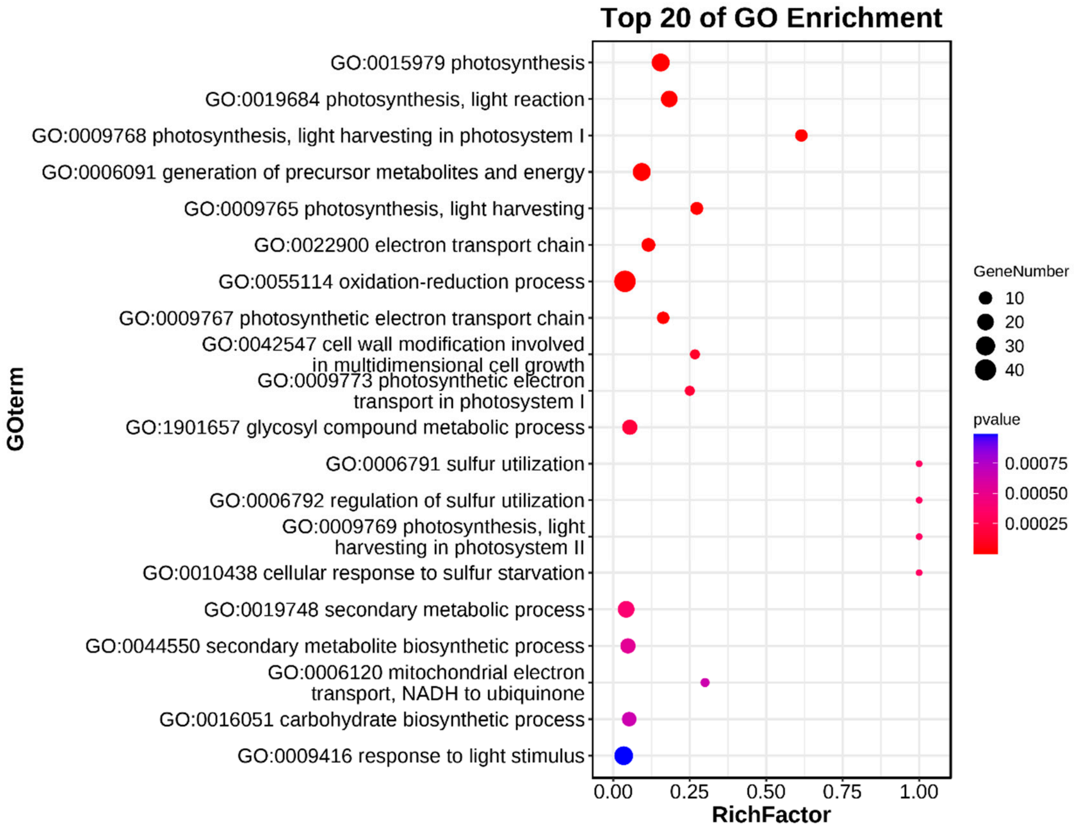

Figure S3 GO term enrichment of the genes with up-regulated expression in roots of ***A. odoratissima***.

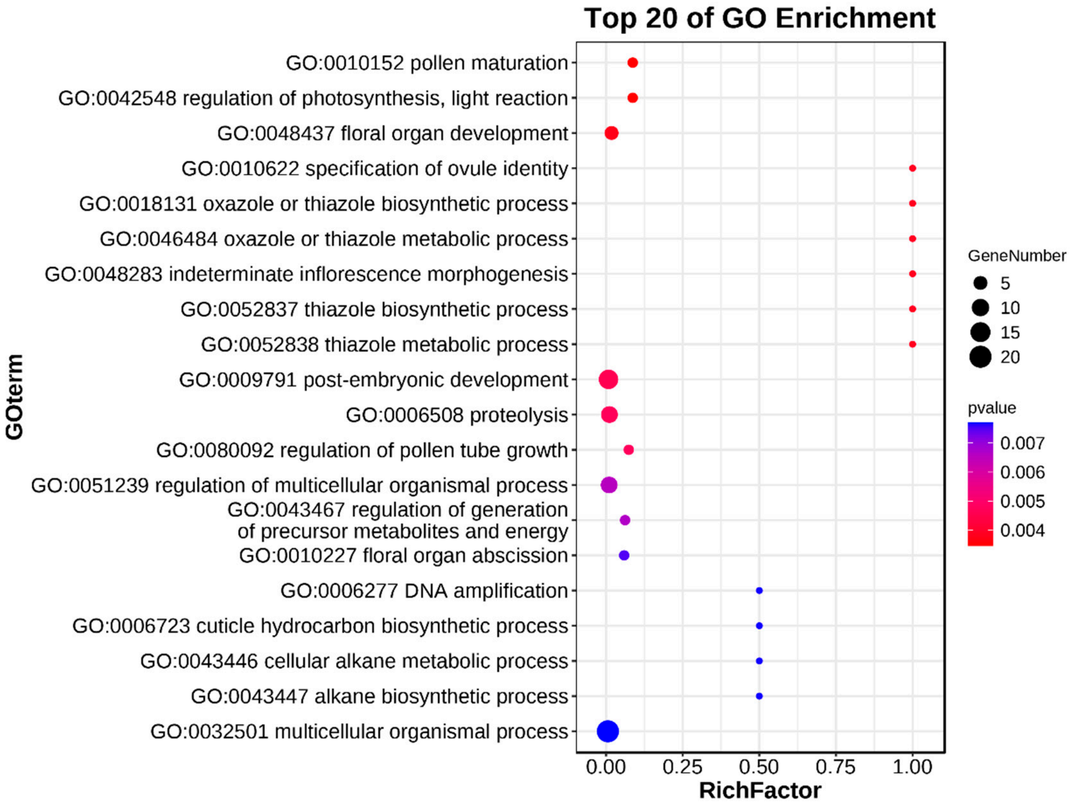

Figure S4 GO term enrichment of the genes with down-regulated expression in roots of ***A. odoratissima***.

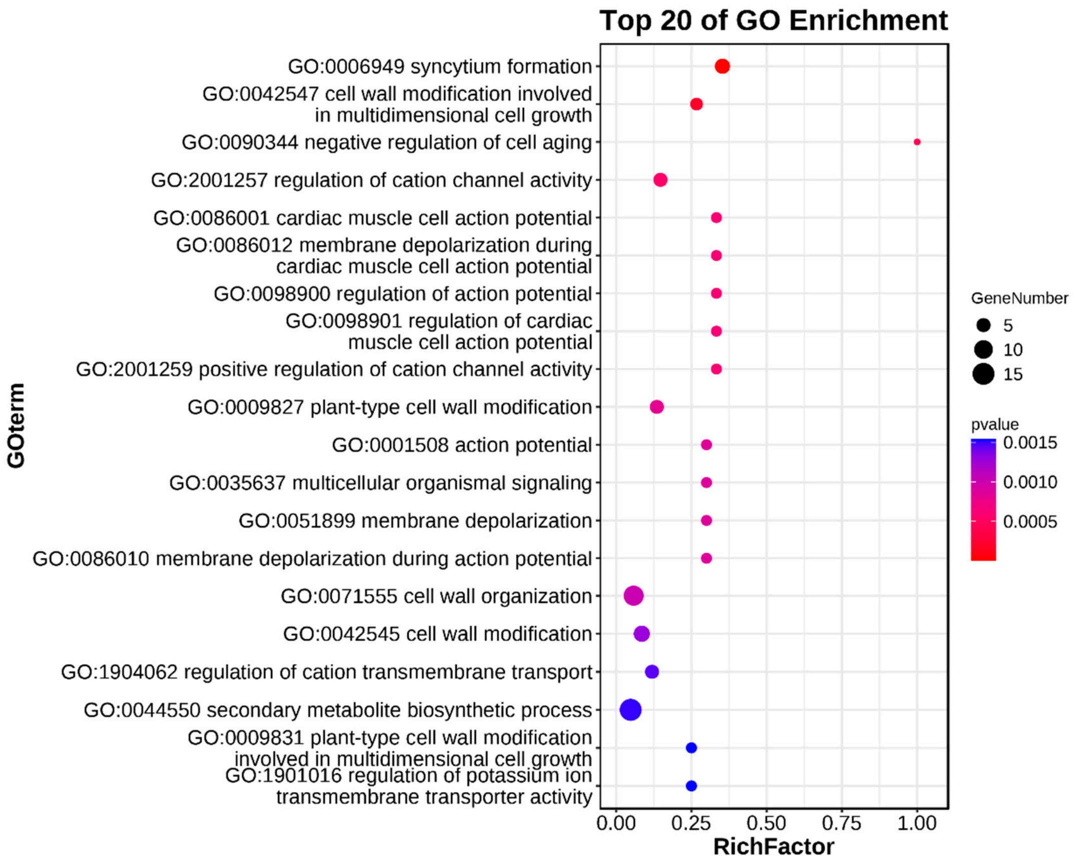

Figure S5 Correlation heat map between different modules and traits in WGNNA analysis.

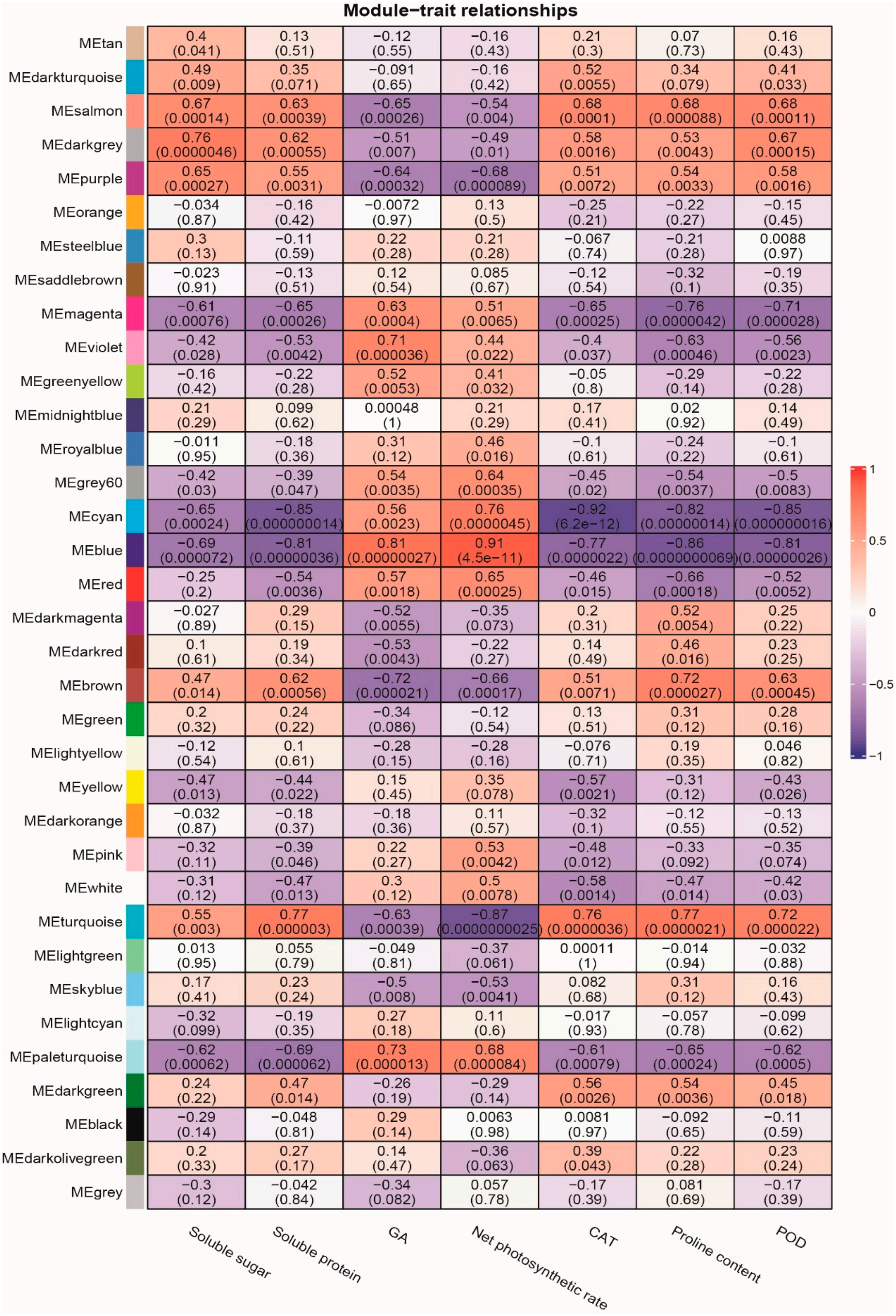

Figure S6 KEGG term enrichment of the genes in blue module.

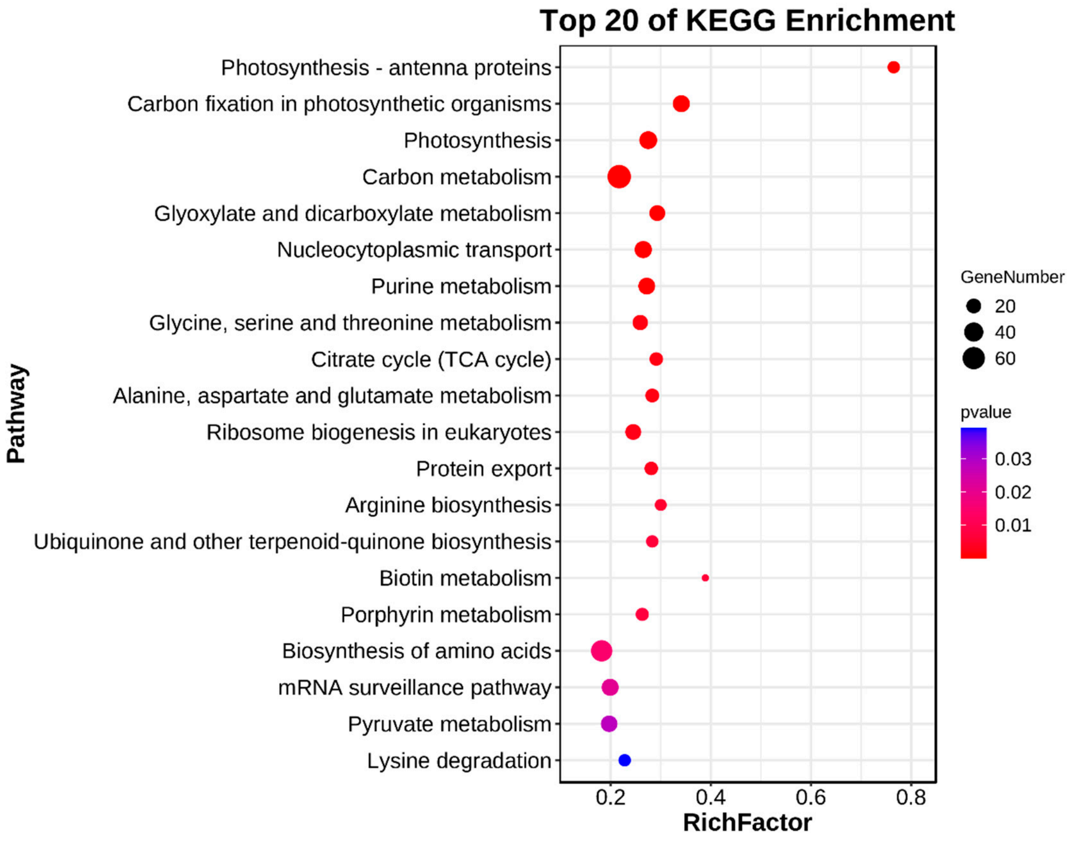

Figure S7 GO term enrichment of the genes in blue module.

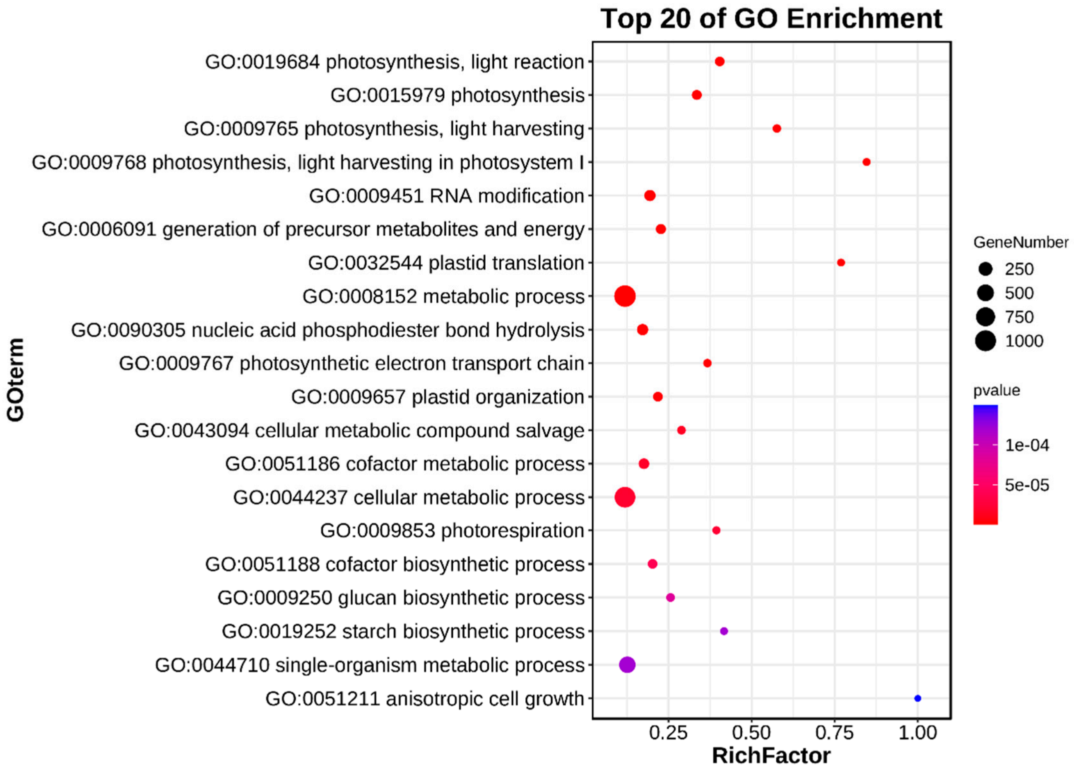

Figure S8 KEGG term enrichment of the genes in cyan module.

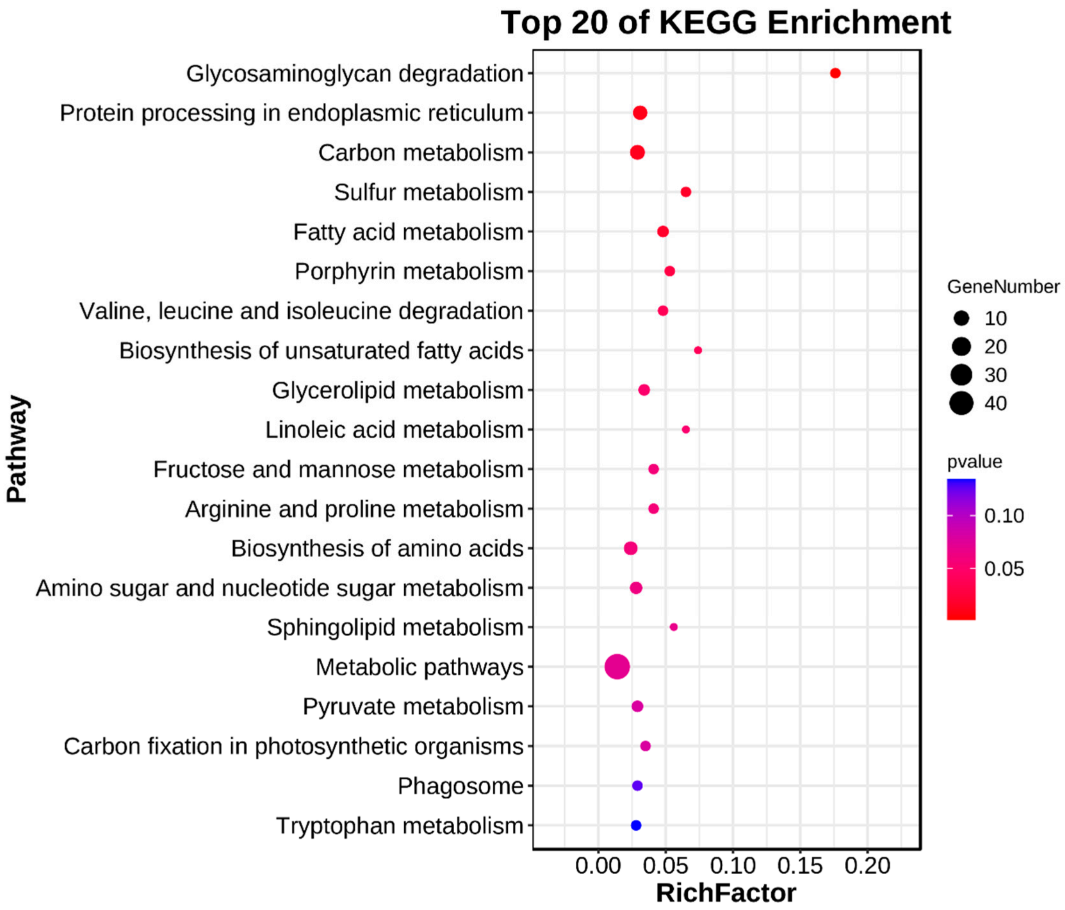

Figure S9 GO term enrichment of the genes in cyan module.

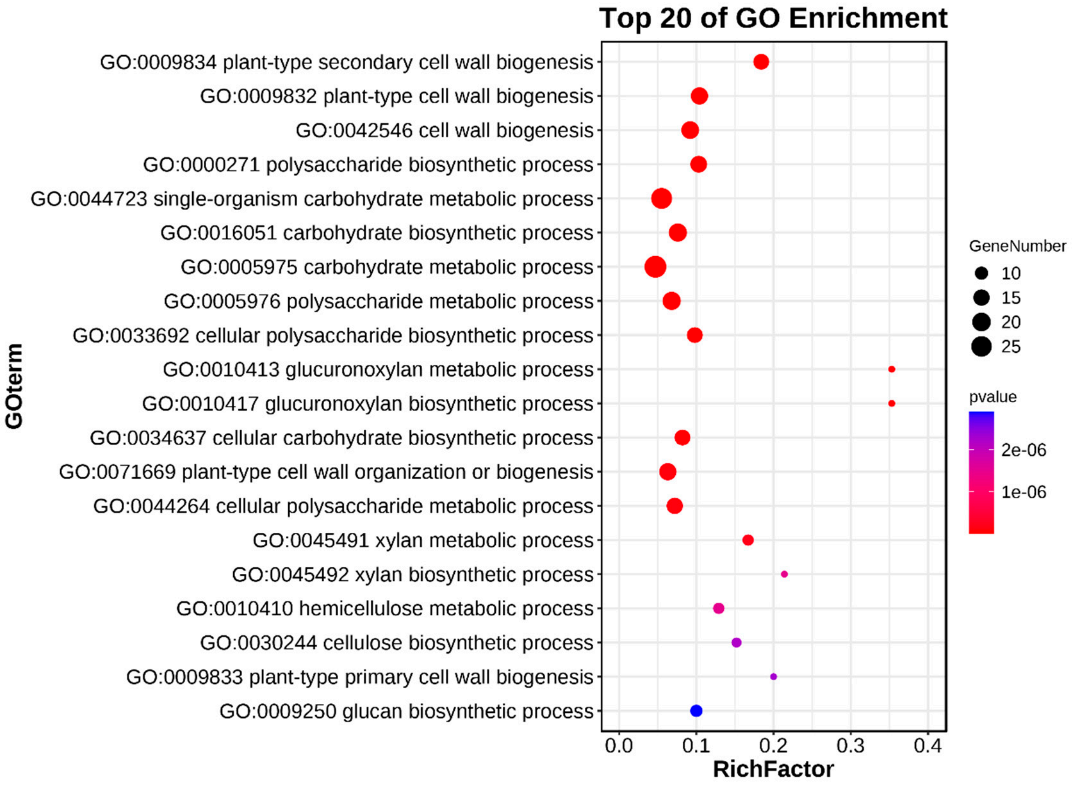

Figure S10 Validation of DEGs by qRT-PCR.

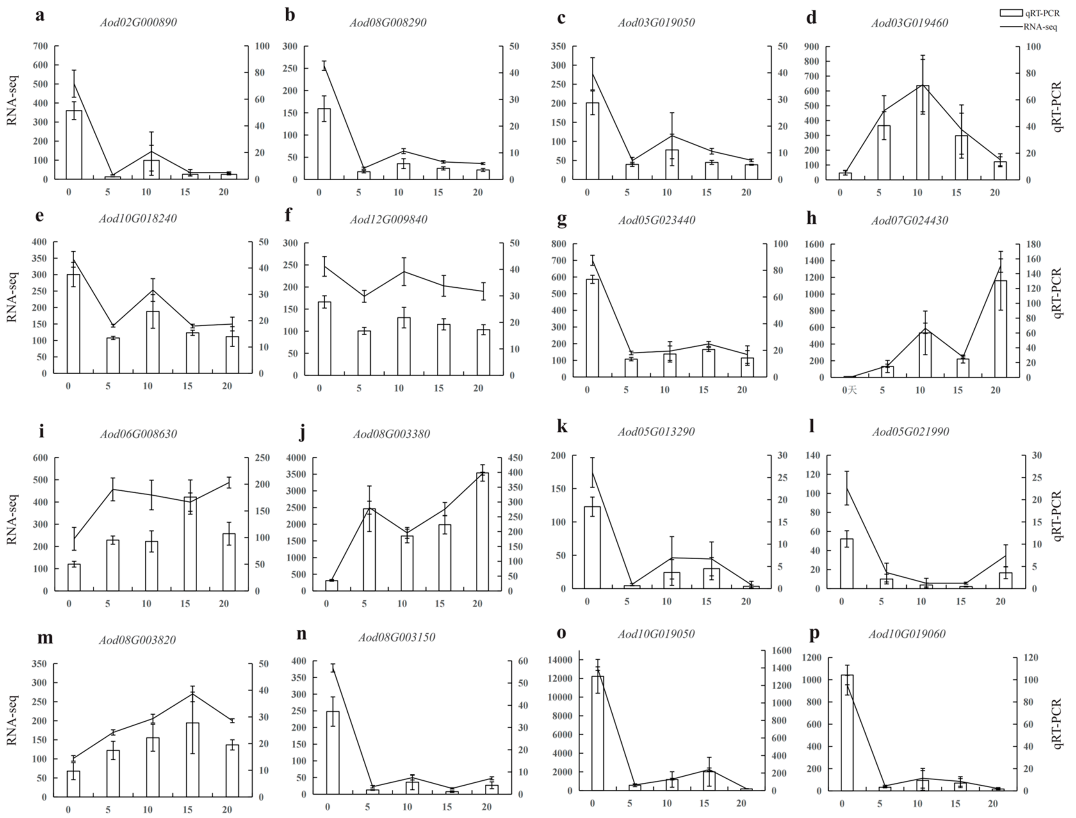

Supplement: Supplementary file 1 [file plants-13-02732-s001.zip › Figure S1-S10.pdf]
