# Supplementary material for: Transcriptome and Metabolome Analyses Reveal the Molecular Mechanisms of Albizia odoratissima’s Response to Drought Stress
Source: Plants (Basel). 2024 Sep 29;13(19):2732. doi: 10.3390/plants13192732 (PMC11478484; doi:10.3390/plants13192732)
Supplement: Supplementary file 1 [file plants-13-02732-s001.zip › Table S1-S2.pdf]

Table S1 Effect of drought days on leaf pulp of *A. odoratissima* seedlings.

	T_0	T_5	T_10	T_15	T_20
Leaf thickness/μm	114.51±11.45a	104.18±3.80a	98.18±6.86a	97.96±5.97a	94.39±8.27a
Upper thickness /μm	14.51±0.79a	17.11±3.58a	14.67±2.11ab	12.23±5.84a	11.71±2.04a
Lower thickness/μm	10.50±1.87a	11.90±2.85a	13.19±0.16a	10.64±3.48b	10.20±2.01a
Palisade tissue thickness/μm	49.19±5.52a	50.08±7.20a	46.69±6.38a	45.06±0.70a	44.12±7.90a
Spongy tissue thickness/μm	38.36±3.05a	31.19±1.39a	31.10±2.19a	30.88±0.32a	29.39±2.30a
The ratio of palisade tissue and pongy parenchyma thickness/μm	45.11±2.63a	40.63±3.05Aa	38.89±3.78Aa	37.97±0.45a	36.76±5.10a
Xylem thickness/μm	29.05±3.55a	25.61±3.80a	24.70±3.08a	26.63±0.13a	23.71±4.98a
Main vein thickness/μm	204.83±38.14a	157.84±21.201a	151.884±5.18a	149.64±6.46b	137.47±7.06b

Table S2 Summary of transcriptome data.

Sample Name	Clean Reads	Clean Base	Read Length	Q20(%)	GC(%)
root-0-1B	24,108,743	7,232,622,900	PE150	97.27	45.69
root-0-2B	24,125,310	7,237,593,000	PE150	97.28	44.68
root-0-3B	24,030,639	7,209,191,700	PE150	97.31	44.98
leaf-0-1A	22,056,247	6,616,874,100	PE150	97.77	45.71
leaf-0-2A	24,111,322	7,233,396,600	PE150	97.28	45.88
leaf-0-3A	24,076,706	7,223,011,800	PE150	97.34	45.67
root-5-CK-1B	24,065,674	7,219,702,200	PE150	97.31	44.87
root-5-CK-2B	24,071,546	7,221,463,800	PE150	97.18	45.03
root-5-CK-3B	24,106,306	7,231,891,800	PE150	97.09	45.49
leaf-5-CK-1B	24,003,026	7,200,907,800	PE150	97.19	47.12
leaf-5-CK-2B	22,644,204	6,793,261,200	PE150	97.36	48.22
leaf-5-CK-3B	24,085,816	7,225,744,800	PE150	97.23	47.3
root-5-1B	24,082,504	7,224,751,200	PE150	97.23	45.41
root-5-2B	24,031,316	7,209,394,800	PE150	97.25	45.66
root-5-3B	24,028,228	7,208,468,400	PE150	97.15	46.15
leaf-5-1A	24,031,677	7,209,503,100	PE150	97.16	45.65
leaf-5-2B	25,365,966	7,609,789,800	PE150	97.18	46.88
leaf-5-3B	27,724,681	8,317,404,300	PE150	98.31	47.62
root-10-CK-1B	24,105,978	7,231,793,400	PE150	97.54	46.54
root-10-CK-2B	24,127,206	7,238,161,800	PE150	97.55	46.67
root-10-CK-3B	22,316,961	6,695,088,300	PE150	97.48	44.98
leaf-10-CK-1A	24,055,786	7,216,735,800	PE150	97.26	45.2
leaf-10-CK-2B	24,077,064	7,223,119,200	PE150	97.61	46.18
leaf-10-CK-3A	24,098,876	7,229,662,800	PE150	97.35	46

root-10-1B	24,009,225	7,202,767,500	PE150	97.55	46.12
root-10-2B	24,012,682	7,203,804,600	PE150	97.5	45.93
root-10-3B	24,104,522	7,231,356,600	PE150	97.45	45.28
leaf-10-1B	24,071,925	7,221,577,500	PE150	97.39	45.18
leaf-10-2A	24,098,248	7,229,474,400	PE150	97.42	47.76
leaf-10-3B	24,111,255	7,233,376,500	PE150	97.42	44.48
root-15-CK-1B	24,116,795	7,235,038,500	PE150	97.56	44.83
root-15-CK-2B	24,141,631	7,242,489,300	PE150	96.52	45.13
root-15-CK-3B	24,013,679	7,204,103,700	PE150	96.65	44.98
leaf-15-CK-1B	24,065,969	7,219,790,700	PE150	97.56	45.39
leaf-15-CK-2B	24,011,115	7,203,334,500	PE150	97.52	45.29
leaf-15-CK-3B	24,069,954	7,220,986,200	PE150	97.47	45.59
root-15-1B	24,112,029	7,233,608,700	PE150	96.64	44.41
root-15-2B	24,003,002	7,200,900,600	PE150	96.58	44.19
root-15-3B	24,028,498	7,208,549,400	PE150	96.56	44.62
leaf-15-1A	24,140,887	7,242,266,100	PE150	97.33	44.81
leaf-15-2A	24,125,975	7,237,792,500	PE150	97.23	45.31
leaf-15-3A	24,090,559	7,227,167,700	PE150	97.19	46.59
root-20-CK-1B	24,091,244	7,227,373,200	PE150	96.48	46.36
root-20-CK-2B	24,043,708	7,213,112,400	PE150	96.71	45.73
root-20-CK-3B	24,071,715	7,221,514,500	PE150	96.33	47.06
leaf-20-CK-1A	24,074,902	7,222,470,600	PE150	97.14	50.77
leaf-20-CK-2B	24,024,057	7,207,217,100	PE150	96.66	45.24
leaf-20-CK-3A	24,093,446	7,228,033,800	PE150	97.21	46.48
root-20-1B	24,072,289	7,221,686,700	PE150	96.47	45.19
root-20-2B	24,128,194	7,238,458,200	PE150	96.52	45.63

root-20-3B	24,086,699	7,226,009,700	PE150	96.76	45.5
leaf-20-1A	24,026,334	7,207,900,200	PE150	98.02	54.08
leaf-20-2A	24,027,948	7,208,384,400	PE150	97.86	55.29
leaf-20-3A	24,119,054	7,235,716,200	PE150	97.87	54.98
